# Effect-Directed Profiling of Monofloral Honeys from Ethiopia by High-Performance Thin-Layer Chromatography and High-Resolution Mass Spectrometry

**DOI:** 10.3390/molecules27113541

**Published:** 2022-05-31

**Authors:** Gertrud E. Morlock, Abera Belay, Julia Heil, Annabel Mehl, Hannelore Borck

**Affiliations:** 1 Institute of Nutritional Science, Chair of Food Science, Justus Liebig University Giessen, Heinrich-Buff-Ring 26–32, 35392 Giessen, Germany; ab.berabelay@gmail.com (A.B.); Julia.Heil@ernaehrung.uni-giessen.de (J.H.); Annabel.Mehl@ernaehrung.uni-giessen.de (A.M.); hhborck@t-online.de (H.B.); 2 Department of Food Science and Applied Nutrition, Addis Ababa Science and Technology University, Addis Ababa P.O. Box 16417, Ethiopia

**Keywords:** HPTLC-direct bioautography, radical scavenging assay, antioxidative assay, antibacterial assay, enzyme inhibition assay, HPTLC–HRMS

## Abstract

Ethiopian honey is used not only as food but also for treatment in traditional medicine. For its valorization, bioactive compounds were analyzed in nine types of monofloral Ethiopian honey. Therefore, a non-target effect-directed profiling was developed via high-performance thin-layer chromatography combined with multi-imaging and planar effect-directed assays. Characteristic bioactivity profiles of the different honeys were determined in terms of antibacterial, free-radical scavenging, and various enzyme inhibitory activities. Honeys from *Hypoestes* spp. and *Leucas abyssinica* showed low activity in all assays. In contrast, others from *Acacia* spp., *Becium grandiflorum,* *Croton macrostachyus*, *Eucalyptus globulus,* *Schefflera abyssinica*, *Vernonia amygdalina*, and *Coffea arabica* showed more intense activity profiles, but these differed depending on the assay. In particular, the radical scavenging activity of *Croton macrostachyus* and *Coffea arabica* honeys, the acetylcholinesterase-inhibiting activity of *Eucalyptus globulus* and *Coffea arabica* honeys, and the antibacterial activity of *Schefflera abyssinica* honey are highlighted. Bioactive compounds of interest were further characterized by high-resolution mass spectrometry. Identifying differences in bioactivity between mono-floral honey types affects quality designation and branding. Effect-directed profiling provides new insights that are valuable for food science and nutrition as well as for the market, and contributes to honey differentiation, categorization, and authentication.

## 1. Introduction

Honey is a complex natural food that has been consumed worldwide since ancient times. It is produced by bees that ingest and concentrate the nectar of flowers. It is an aqueous but viscous concentrate of different saccharides, mainly glucose and fructose, produced without food additives or technical adjuvants. Apart from the saccharides, honey also contains phytochemicals (derived from the plant nectar taken up by the bees), enzymes (added by the bees, when the nectar is stored in the honey stomach during transport), and metabolites (resulting from digestive metabolic processes during transport and concentration during mouth-to-mouth transport in the beehive). In this way, the flower nectar changes and matures into honey. In the case of honeydew, sweet insect excretes (mainly from aphids, leaf fleas, and cicadas) are collected and further metabolized by bees. The minor components therefore change depending on the honeybee species, geographical origin, and available floral nectar or insect source. Honeys contain, among others, amino acids, organic acids, phenolic acids and further (poly) phenolic compounds, flavonoids, carotenoid-like substances, terpenes, Maillard reaction products such as 5-(hydroxymethyl)furfural, vitamins (ascorbic acid, tocopherols, carotenes), minerals, aroma compounds, pigments, waxes, pollen, and enzymes [1]. Honey is also used in traditional medicines due to the bioactive compounds present in the complex mixture [2,3].

In Ethiopia, different types of monofloral blossom honey as well as honeydew honey are produced [4,5], which is important for the bee economy of the country [6]. Products were reported from *Acacia* species, *Becium grandiflorum*, *Croton macrostachyus*, *Eucalyptus globulus*, *Hypoestes* species, *Leucas abyssinica*, *Schefflera abyssinica*, *Syzygium guineense*, and Harenna forest [7,8,9,10]. These were analyzed according to different standardized protocols described in the *Codex Alimentarius* by the International Honey Commission and Association of Official Analytical Chemists [11]. Ethiopian monofloral blossom honeys have a moisture content of 14–21% and contain mainly fructose (35–43%), glucose (29–37%), sucrose (1.1–2.8%), maltose (0.6–2.0%), turanose (0.3–1.7%), and isomaltose (0.0–1.5%). Three amino acids were largely found, i.e., phenylalanine (5–119 mg/100 g), proline (16–74 mg/100 g), and aspartic acid (8–22 mg/100 g). Their 5-(hydroxymethyl)furfural content ranged from 0.5 to 3.4 mg/kg, diastase activity ranged from 4.9 to 13.5 Schade units, and invertase activity ranged from 11.6 to 36.5 IN [7,8,9,10]. In addition to determining basic properties, there is growing interest in fingerprinting techniques that are associated with characteristic constituents for authentication [12,13,14,15,16]. Information on the bioactivity of honey is also gaining interest [17,18,19,20] to add value to food beyond the purpose of sweetening. For example, the antioxidative effect and total phenolic content of *Eucalyptus* honey from Portugal [16] or *Schefflera abyssinica* honey from Ethiopia [10] was measured as a sum parameter, and the characteristic profile of different flavonoids of *Eucalyptus* honeys from Southern Europe was determined [15].

By combining a chromatographic separation with a non-target assay, it is possible to truly measure the activity of bioactive compounds and, in particular, to differentiate opposing effects. This would provide more understanding of honey quality than is possible with simple in vitro assays (providing only sum parameters for complex samples) or nuclear magnetic resonance spectroscopy (without actual proof of activity). Such a non-target effect-directed profiling was shown for plant-based food [21,22,23,24], but has not yet been applied for Ethiopian honey samples. In this study, a profiling method was developed to detect individual bioactive compounds in honey. It was considered important to recognize differences in bioactivity between monofloral honeys, which can affect quality designation and branding. In particular, the non-target analysis of bioactive compounds could provide new insights, also regarding differentiation, categorization, and authentication of the types of honey. Nine types of monofloral Ethiopian honey were screened side by side for radical scavenging, antimicrobial, and enzyme inhibition activities, using high-performance thin-layer chromatography combined with multi-imaging and planar effect-directed assays (HPTLC−UV/Vis/FLD−EDA) and (heated) electrospray high-resolution mass spectrometry (HPTLC–HESI-HRMS) for further characterization of zones of interest.

## 2. Results and Discussion

### 2.1. Development of the Physico-Chemical Profiling

Ethiopian honey types originating from herbs, shrubs, and trees were collected at the farm gate, directly from the beekeepers in the honey-harvesting period. The honey colors were mostly brown but also bright, ochre, and blackish (Table 1). The samples were proven via pollen analysis by melissopalynology [4,5,10] and categorized into the respective monofloral honey types. The honey samples were freed from saccharides by solid-phase extraction using a spherical, hydrophobic polystyrene–divinylbenzene adsorbent and methanol as eluent. Different mobile-phase mixtures were tested for separation of the extracts on HPTLC silica gel 60 F_254_ plates. A mixture of ethyl acetate and methanol in the ratio of 3:2, *V*/*V,* was found to be suitable, since it spread the components along the migration distance. No particular analytes were selected at this stage for non-target profiling. The parallel separation of honey samples took only 14 min. The detection via multi-imaging at white light illumination (Vis), 254 nm (UV), and 366 nm (FLD) took 3 min.

**Figure 1:**
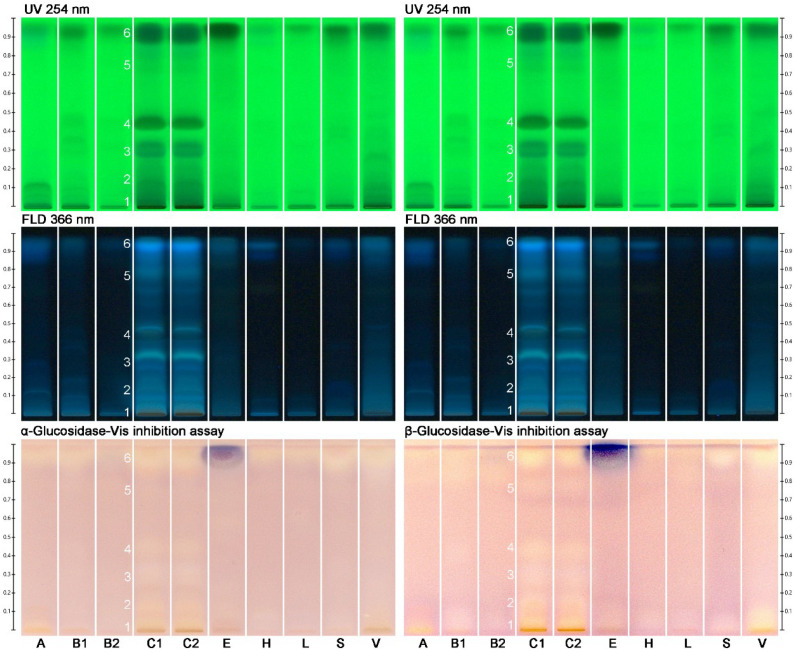
Physico-chemical profiling at UV 254 nm and FLD 366 nm of 10 monofloral honey extract samples (assigned as in Table 1; 3 µL/band each) on HPTLC silica gel 60 F_254_ plates with ethyl acetate–methanol 3:2, *V*/*V*, as well as effect-directed profiling via the α- and β-glucosidase inhibition assays, detecting the glucosidase inhibiting zones **1**−**6** at white light illumination (Vis).

Next, the different monofloral honey sample extracts (Table 1) were subjected to physico-chemical profiling by HPTLC−UV/Vis/FLD to obtain information on the differences in their phytochemical patterns and their diversity. Visible zones were not observed under white light illumination (data not shown). The honey extracts from tree or shrub sources, *i.e.*, *Acacia* ssp. (A), *Croton macrostachyus* (C)*, Schefflera* *abyssinica* (S), *Eucalyptus globulus* (E), and *Vernonia* *amygdalina* (V), showed comparatively more UV-active (Figure 1, UV 254 nm chromatogram) and bluish fluorescent substances (FLD 366 nm chromatogram) than honey extracts from herb sources, *i.e.*, *Becium grandiflorum* (B), *Hypoestes* ssp. (H), and *Leucas abyssinica* (L). Among these Ethiopian honeys, *Croton macrostachyus* was richest in compounds. Two additional derivatization reagents were tested, since derivatization can readily be performed on the planar chromatogram and provides additional helpful information for further characterization [24]. However, the derivatization using the natural product reagent for the detection of flavonoids did not show additional fluorescent zones at FLD 366 nm at the given amounts applied, whereas when using the primuline reagent for the detection of lipophilic substances, faint blue fluorescent zones were observed at the start zone of *Hypoestes* ssp. as well as in the solvent front for some samples (data not shown). These results and the impression of the samples obtained by this physico-chemical profiling were satisfactory in informative value to start the non-target effect-directed profiling.

### 2.2. Effect-Directed Profiling of Nine Different Types of Monofloral Honey

**Figure 2:**
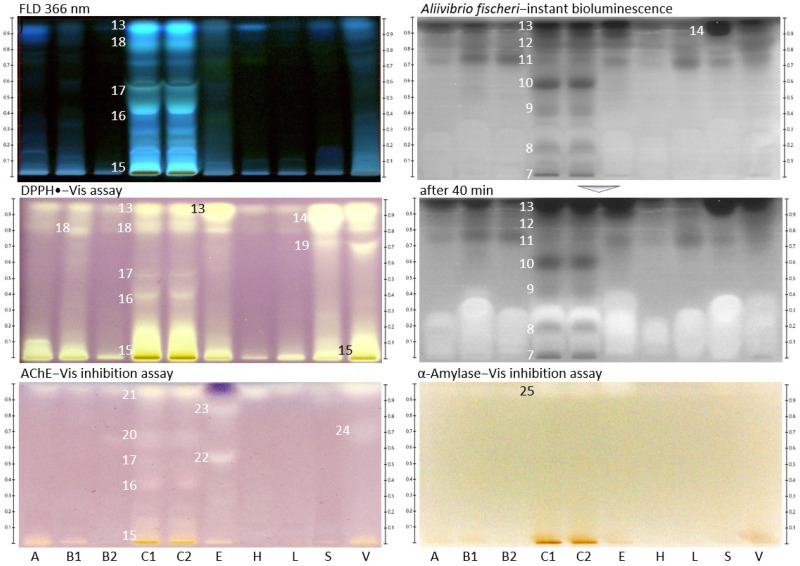
Chromatogram at FLD 366 nm and respective effect-directed profiling of 10 monofloral honey extract samples (assigned as in Table 1; 5 µL/band for *Aliivibrio fischeri* bioassay, others 3 µL/band each) developed on HPTLC silica gel 60 F_254_ plates with ethyl acetate–methanol 3:2, *V*/*V*, detected via the *Aliivibrio fischeri* bioassay (bioluminescence recorded as greyscale image instantly and after 40 min) as well as DPPH•, AChE, and α-amylase inhibition assays (white light illumination, Vis), revealing the bioactive zones **7**–**25**.

The same chromatogram was prepared several times and subjected to six different planar effect-directed assays targeting antibacterials (against Gram-negative bacteria), 2,2-diphenyl-1-picrylhydrazyl (DPPH•) scavengers (antioxidants), and inhibitors of acetylcholinesterase (AChE), α-glucosidase, β-glucosidase, and α-amylase. In the case of the immersion technique, minor zone shifts (1−2 mm) were observed, as reported in [21], which needed to be taken into account for zone matching on the different images. The effect-directed profiling via HPTLC−UV/Vis/FLD−EDA (Figure 1 and Figure 2) showed characteristic fingerprints of the nine different types of monofloral honey that differed in number and position as well as the intensity of the bioactive zones. Similar to the physico-chemical profiling, honey extracts from tree or shrub sources showed comparatively more bioactive reactions than honey extracts from herb sources, and *Croton macrostachyus* was richest in bioactive compounds across all assays. Comparable effect-directed profiles were obtained for the two *Croton macrostachyus* honey extract samples, which was expected because it was a repeated analysis of the same sample (Table 1). This highlighted the repeatability of the profiling. In contrast, the two *Becium grandiflorum* honey extracts, which were collected at two different sites in Ethiopia, showed the same activity pattern in the same assays but differed strongly in intensity. This clearly shows that nominally, the same monofloral honey originating from different sites can differ in the intensity of the inherent bioactive zones.

The α- and β-glucosidase inhibition autograms (Figure 1) showed weak to moderate inhibition of the α- and β-glucosidase when 9 µL/band (equivalent to 90 mg honey) were applied. Almost the same zones were evident in both enzyme assays, although the inhibition of β-glucosidase was stronger. *Croton macrostachyus* showed the most pronounced responses and revealed glucosidase-inhibiting compounds **1**–**6** (evident as colorless or bright zones) not only near the solvent front and in the start zone, as evident in other scrub/tree honey samples, but also in further zones in between. The honey extracts from herb sources (*Becium grandiflorum*, *Hypoestes* ssp., and *Leucas abyssinica*) were comparatively weaker in the glucosidase-inhibiting response. The UV 254 nm and FLD 366 nm chromatograms (Figure 1) of both glucosidase inhibition assays highlight the good reproducibility of the profiling.

Several compounds that were active against Gram-negative bacteria were detected as dark zones in the HPTLC-*Aliivibrio fischeri* bioautogram (Figure 2). The bioluminescence image was monitored over a period of 40 min, wherefrom the instantly recorded bioautogram is depicted as well as the one after 40 min. The latter bioautogram was found to be more important since it indicated strong longer-lasting effects. All investigated honey extract samples showed antibacterial activity; however, they were quite different in number and intensity of the individual responsible compounds. *Croton macrostachyus* honey extract revealed the darkest zones, numbered **7**–**13** (Figure 2). The most pronounced antibacterial zone (**14,** near the solvent front) was evident in *Schefflera* *abyssinica* honey extract, which was instantly detected and indicated a strong acute effect. Interestingly, the longer the monitoring period, the more bright zones were found, as evident in the bioluminescence image recorded after 40 min. Observing changing effects and distinguishing between individual enhancing and darkening effects is very important for understanding. Only then can one recognize and distinguish whether it is an acute or gentle and slower-reacting or even reversing (bacteria do recover) or a strong long-lasting effect. This provides hints for bacteriostatic or bactericide mechanisms of action. Such a deep understanding cannot be achieved with the commonly used in vitro tests because these provide an incorrect sum parameter value for opposing effects in complex samples such as honey (Figure 2, *Aliivibrio fischeri* bioautogram after 40 min; the opposing effects increased in intensity over time, images between 0 and 40 min are not depicted).

The radical scavenging (antioxidative) activity was investigated using the HPTLC-DPPH• assay. All tested honey extract samples showed radical scavenging activity (visible as a yellow zone against a purple background) with a varying intensity and number of active zones (Figure 2, zones **15**–**19**). In the DPPH• autogram, the horizontal intensity pattern for zones **13** and **14** (both near the solvent front) across the samples was comparable to that in the *Aliivibrio fischeri* bioautogram. Hence, the zones were found to be the same (same numbers as before).

All investigated honey extract samples revealed an inhibition of the AChE (visible as a colorless or bright zone against a purple background) near the solvent front (Figure 2, zone **21**). In particular, *Croton macrostachyus*, *Eucalyptus globulus*, and *Vernonia* *amygdalina* showed AChE inhibiting zones **15**–**17** and **20**–**24**. Above zone **23,** the same lilac zone was observed as in the previous anti-glucosidase assays (Figure 1). It was proven (using the same workflow but without enzyme) to be a color reaction with the Fast Blue B salt and assumed to be a phenolic compound. It is known that reactions of sample compounds with substrates/chromogenic reagents can occur [25].

Honey extracts from *Croton macrostachyus* and *Eucalyptus globulus* in particular showed moderate inhibiting effects on the α-amylase (visible as a colorless or bright zone against a yellow background)*,* whereas it was weaker in the response for *Schefflera abyssinica* and *Vernonia* *amygdalina*. These α-amylase-inhibiting activities were caused by a more lipophilic compound near the solvent front (Figure 2, zone **25**). Comparing the different assay results, it could have been the same substance as in zone **6,** but it could not be confirmed for *Eucalyptus globulus* due to the coeluting lilac zone, as discussed. Hence, a new number was given. Note that bioactive zones were assigned to a previous number based on similarity to a previous horizontal activity pattern (at the same *hR*_F_ position across the different samples) but also newly numbered, if there was no pattern in common (or it was uncertain). The numbering of bioactive zones was also transferred to the images of the physico-chemical profiling, which gave an additional hint regarding native spectral properties as UV-absorber or fluorophore (e.g., Figure 1, zone **6**), or in case the horizontal pattern did not match, it was helpful for orientation (e.g., Figure 2, zone **13**). Hence, bands numbered in the physico-chemical profiling are not necessarily meant to be bioactive zones.

In agreement with our results (high bioactivity of *Croton macrostachyus*), high antioxidant and antibacterial bioactivity was shown for another *Croton* species (*Croton lechleri*) of the same Euphorbiaceae family, whose essential oil was discussed as a new functional food ingredient [26]. The intensities of the antioxidative zones in the autogram were in accordance with results of a photometric method reporting ascorbic acid equivalents (AAE) [27]. Therein, the honey extract of *Hypoestes* ssp. showed the weakest activity, with 9 mg AAE/kg, whereas the *Croton macrostachyus* honey extract showed the highest activity, with 166 mg AAE/kg, followed by the *Vernonia* *amygdalina* honey extract, with 149 mg AAE/kg. The same ranking was evident in our DPPH• autogram (Figure 2). The honey extracts from herb sources (*Becium grandiflorum*, *Hypoestes* ssp., and *Leucas abyssinica*) were comparatively weaker in the radical scavenging response. Interestingly, the antioxidant activity of monofloral honeys could not be correlated to high values of flavonoids therein [17].

### 2.3. Effect-Directed Profiling within the Same Type of Monofloral Honey

**Figure 3:**
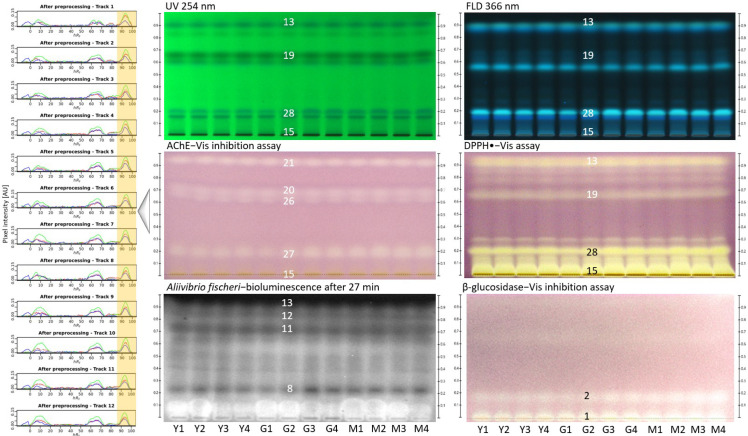
Chromatograms at UV 254 nm and FLD 366 nm and respective effect-directed profiling of 12 *Coffea arabica* honey extract samples (2 µL/band each; samples 1−4 collected at different sites in the Yayu (Y), Goma (G), and Mana (M) regions, Table 1 developed on HPTLC silica gel 60 F_254_ plates with ethyl acetate–methanol 3:2, *V*/*V*, detected via the DPPH• radical scavenging assay, AChE and β-glucosidase inhibition assays (white light illumination, Vis), and *Aliivibrio fischeri* bioassay (bioluminescence after 27 min depicted as greyscale image); AChE inhibition autogram evaluated via quanTLC software to measure the variation of the pixel signal intensity of zone **21** (framed in yellow).

Ethiopian coffee honey is attracting special interest and was also subjected to effect-directed profiling. Twelve *Coffea arabica* honeys were collected from the Ethiopian regions of Yayu (Y), Goma (G), and Mana (M), each at four different locations (Table 1). The chromatograms at UV 254 nm and FLD 366 nm of the 12 *Coffea arabica* honey extract samples (2 µL/band each, equivalent to 20 mg honey) showed a characteristic pattern that was very similar between the 12 samples (Figure 3) but quite different to the previous monofloral honey extract samples (Figure 1 and Figure 2). Each respective effect-directed profiling (via the DPPH• radical scavenging assay, AChE and β-glucosidase inhibition assays, and *Aliivibrio fischeri* bioassay) showed highly comparable bioactivity patterns between the 12 samples. In particular, AChE inhibition and DPPH• radical scavenging activities were determined in the *Coffea arabica* honey extract samples. These bioactivity profiles of *Coffea arabica* honey were quite different when compared to those of *Croton macrostachyus,* which was the most active candidate in the effect-directed profiling of the previous monofloral honeys.

As an example, the AChE inhibition autogram was evaluated using the open-source quanTLC software [28]. The variation of the activity of the AChE inhibiting compound zone **21** across all 12 different *Coffea arabica* samples (Figure 3, framed in yellow) was 6.3% (*%RSD*, *n* = 12, green channel signal). This variance is in agreement with the visual impression in the autogram, which showed similar AChE inhibitory responses for zone **21** across the samples. This example of digital evaluation of the autogram shows the potential to use the enzymatic or biological responses for drawing quantitative conclusions about the activity depending on the region or harvest time.

### 2.4. Bioactivities Found in Monofloral Honeys

The main bioactivity responses obtained across the investigated honey samples are summarized in Table 2. It indicates that *Coffea arabica* (Table 1, Y, G, M; here YGM) and *Croton macrostachyus* (C) honeys were richest and strongest in bioactivity. The two are the most promising candidates among the investigated Ethiopian monofloral honey types, taking into account the investigated effect-directed profiles.

### 2.5. Optional On-Surface Extraction and Separation on the Same Plate

For non-target profiling, sample preparation should be minimal, as compounds may be lost or altered at each step. In addition, the solid-phase extraction step is time-consuming and expensive. Hence, a simpler solution was targeted. Sample preparation could be performed on the surface of the adsorbent [29,30]; however, the high saccharide content might restrict the sample load. This challenge was explored exemplarily for three different monofloral honeys, i.e., *Becium grandiflorum*, *Coffea arabica*, and *Vernonia amygdalina* honeys. Different dissolution/dilution solvents for the honeys were compared with regard to the saccharide load on the adsorbent. As a compromise between saccharide load and loss of polar compounds, methanol was selected. The methanolic honey suspension was only ultrasonicated and centrifuged. Each supernatant (25 µL) was applied as an area of 8 mm × 10 mm on the HPTLC silica gel 60 F_254_ plate. The focusing of the applied area to a sharp start band was studied using single solvents or solvent mixtures, also with small portions of formic or acetic acid (Table 3). 

It was interesting to observe how the different solvent polarities migrated through the heavy saccharide load on the adsorbent. For example, methanol or ethanol, either solely or at a higher portion in the mixture, caused an A-shaped zone distortion. As another example, a V-shaped zone distortion was caused by addition of formic acid when focusing with ethanol–formic acid–water 7:1:2 (*V*/*V*/*V*). These zone deformations were reproducible.

For separation on the HPTLC silica gel 60 F_254_ plate, solvent systems were investigated based on polar–acidic, polar–basic, and polar-to-mid-polar properties (Table 3). As an example of a combined on-surface extraction and separation on the same plate, the start areas were focused twice with acetonitrile–ethanol–ammonia 8:1:2, *V*/*V/V,* up to 30 mm, and after, the plate was cut at 15 mm and developed with toluene–ethyl acetate–formic acid–water 1.6:7:0.8:0.6, *V*/*V/V*/*V*, up to a migration distance of 70 mm (Table 3). As another even simpler example, the start areas were just separated with toluene–ethyl acetate–formic acid–water 2:6:1:0.6, *V*/*V/V*/*V*. However, still compounds were retained at the start area. These attempts showed that on-surface extraction and separation on the same plate has potential, but is challenging due to the high saccharide load.

Further targeted optimization for selective front elution and thus separation of polar compounds (which are currently still trapped) from the saccharide-rich start area is necessary for this new idea of on-surface extraction and separation in the field of fast honey analysis.

### 2.6. Characterization of Selected Bioactive Compounds by HPTLC–HESI–HRMS

**Figure 4:**
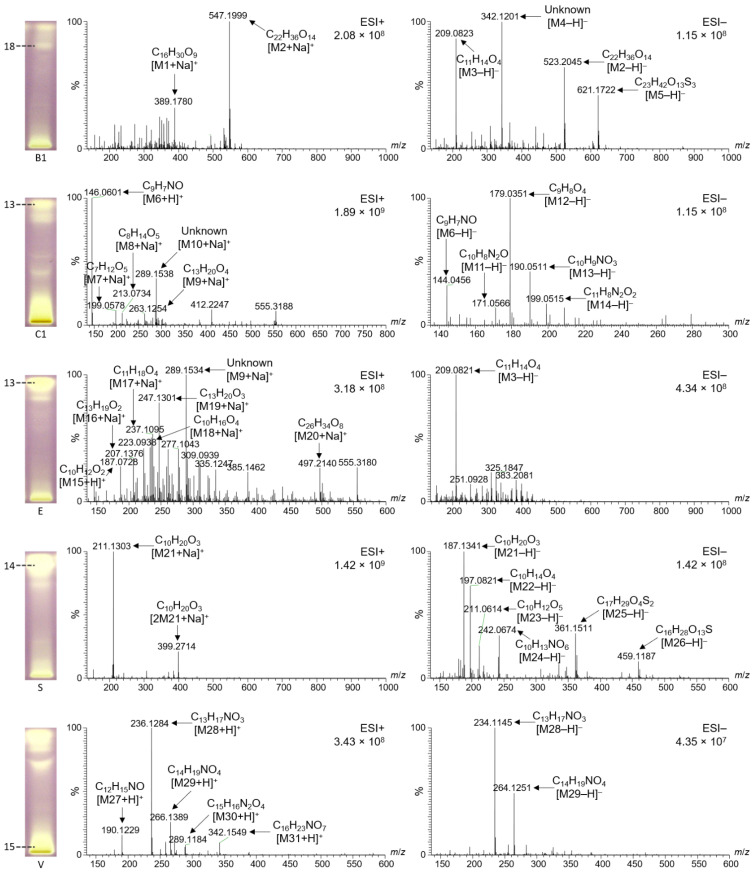
HPTLC–HESI–HRMS spectra in the positive and negative ionization mode of prominent bioactive zones detected via the DPPH• radical scavenging assay (for orientation, zones are marked as in Figure 2) of different monofloral honeys (IDs below the chromatogram stripe, assigned as in Table 1).

**Figure 5:**
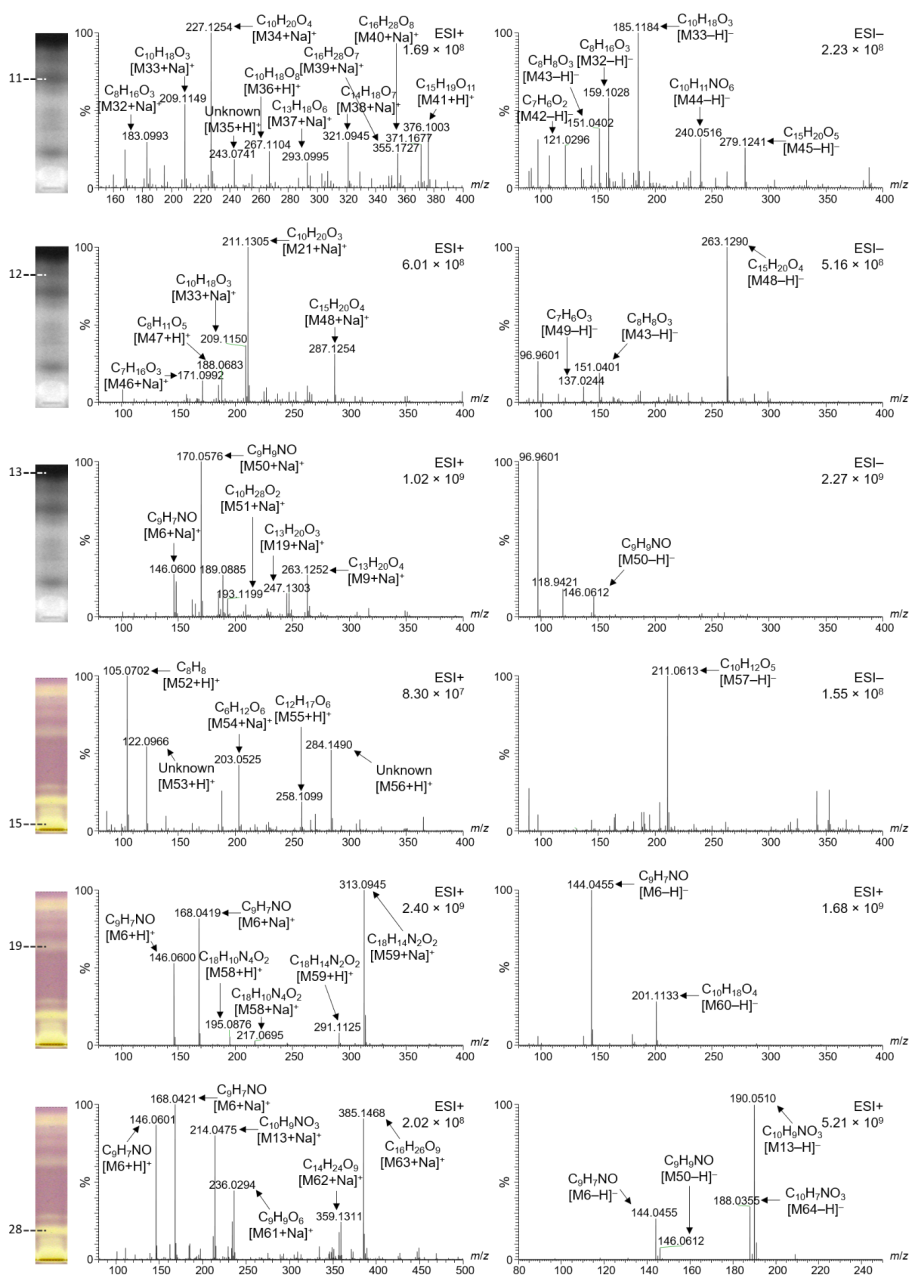
HPTLC–HESI–HRMS spectra in the positive and negative ionization mode of prominent bioactive zones of *Coffea arabica* honey detected via the *Aliivibrio fischeri* bioassay or DPPH• radical scavenging assay (for orientation, zones are marked as in Figure 3).

**Figure 6:**
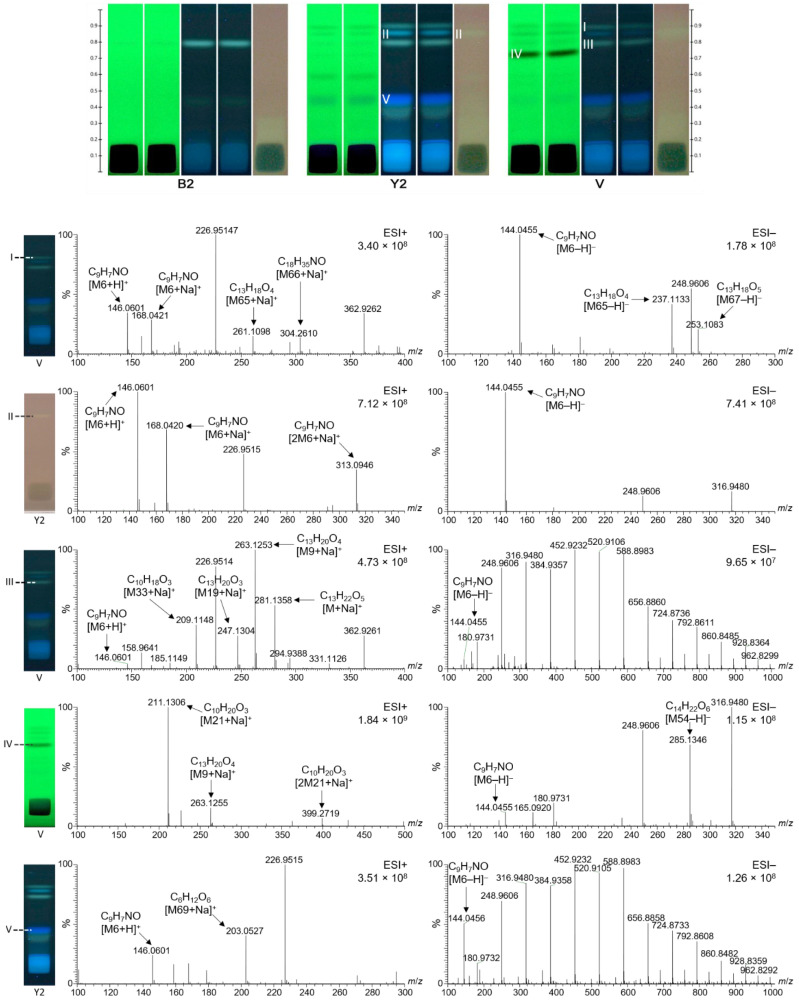
On-surface extraction and separation on the same plate: HPTLC chromatograms of *Becium grandiflorum* (B2), *Coffea arabica* from Yayu (Y2), and *Vernonia amygdalina* (V) honey extracts (25 µL each, note that here the start area focusing was skipped) at UV 254 nm, FLD 366 nm, and at white light illumination after the DPPH• scavenging assay, separated on HPTLC silica gel 60 F_254_ plates with toluene–ethyl acetate–formic acid–water 2:6:1:0.6, *V*/*V/V*/*V,* and HPTLC–HESI–HRMS spectra in the positive and negative ionization mode of selected zones (for orientation, marked with Roman numerals, as the separation system is different to previous ones).

Prominent bioactive zones of the different monofloral honey extracts (Figure 4 and Figure 5) were chosen for further characterization by HPTLC–HESI-HRMS. In addition, honey zones that were fluorescent at FLD 366 nm after on-surface extraction and separation were recorded by HPTLC–HESI-HRMS (Figure 6). Each elution position is marked in Figure 4, Figure 5 and Figure 6 according to the numbering in Figure 1, Figure 2 and Figure 3 for orientation purposes, but should not be misunderstood as equivalent to the actual bioactive zones. For example, radical scavenging zone **13** showed significant differences in the mass signals for *Croton macrostachyus* and *Eucalyptus globulus* at the same elution position, indicating different constituents in honeys of different plant origins. In such a case, zones detected at the same *hR*_F_ position in different samples cannot be assigned to the same number. Hence, different monofloral honeys can show different mass signals even for the same *hR*_F_ positions on the plate, since the bees had collected it from different floral sources, whose secondary metabolites differ. Note that the separation system chosen for on-surface extraction (Figure 6) completely differs from the separation system used for the samples purified using solid-phase extraction (Figure 4 and Figure 5). Thus, the same zones cannot be expected to be at the same *hR*_F_ position, *i.e.*, compound zones do not correspond to previous positions on the plate and mass signals should be different. Hence, these recorded zones were marked with Roman numerals to make evident this difference between solid-phase extraction and on-surface extraction.

Corresponding molecular formulas were assigned to the mass signals obtained (Table 4, Figure 4, Figure 5 and Figure 6). Most zones revealed complex mass signal patterns, especially in the positive ionization mode, resulting in 69 different analytes. These predominantly contained only oxygen as a heteroatom. The analytes closer to the start zone (Figure 4, zone **15**, and Figure 5, zones **19** and **28**) contained nitrogen, whereas some analytes also contained sulfur (Figure 4, zones **14** and **18**). In general, the solid-phase extracts of the honeys (Figure 4 and Figure 5) showed many more different mass signals than the on-surface extracts, which supports the assumption that most compounds were still trapped at the start area due to the high saccharide load.

Some of the bioactive compounds found were matched to possible structures already described for other types of honey in the literature (Table 4). For example, the very prominent analyte M6 was assigned the molecular formula C_9_H_7_NO, which could tentatively be matched to 2-hydroxyquinoline (Table 4). It was detected in all zones of the on-surface extracts (Figure 6), either in positive mode as *m/z* 146.0601 [M6 + H]^+^ and *m/z* 168.0420 [M6 + Na]^+^ or in the negative mode as *m/z* 144.0455 [M6 − H]^−^. It was detected not only in the on-surface extracts, but also in the solid-phase extracts. The corresponding mass signals were distributed over the whole length of the track; thus, it was considered to be a strongly tailing compound. It can be assumed that the slightly brighter color (when compared to the purple background) over the length of all tracks in the effect-directed profiling via the DPPH• assay (Figure 2 and Figure 3) was caused by C_9_H_7_NO. Thus, M6 could also contribute to a synergistic radical scavenging effect with other compounds, as observed in other studies [22]. Interestingly, the mass signals of C_9_H_7_NO were not found in all zones. For example, a comparison of zone **13** from *Croton macrostachyus* and *Eucalyptus globulus* (Figure 4) showed no corresponding mass signals in *Eucalyptus globulus*, but very intense signals in *Croton macrostachyus*, which was considered to be caused by signal suppression by the other analytes. In zone **13** from *Coffea arabica* (Figure 5), not only C_9_H_7_NO but also C_9_H_9_NO was found in the positive and negative ionization mode, which was considered to be related to M6 because it contained one double bond fewer.

Another very intense signal at *m/z* 211.1303 [M21 + Na]^+^ was found in zone **14** from *Schefflera abyssinica* (Figure 4), whereas other related compounds were present in zones **11**, **12**, and **17** from *Coffea arabica* (Figure 5) and in zones **III** and **IV** from *Vernonia amygdalina* (Figure 6). M21 was assigned to the molecular formula C_10_H_20_O_3_ and was tentatively matched to hydroxy decanoic acid (Table 4). The mass signal at *m/z* 263.1254 [M9 + Na]^+^ was assigned to C_13_H_20_O_4_. M9 and some related compounds were detected in zone **13** from *Croton macrostachyus*, *Eucalyptus globulus* (Figure 4), and *Coffea arabica* (Figure 5), and in zones **III** and **IV** from *Vernonia amygdalina* (Figure 6). In most cases, the HRMS spectra were free from residual sugars of the honey matrix, except for zone **15** close to the application zone (Figure 5) and zone **V** at *hR*_F_ 45 in the on-surface extracts (Figure 6), both at similar intensity. This clearly verified the high potential of on-surface extraction to sufficiently remove matrix interferences.

### 2.7. Limitation of the Developed Effect-Directed Profiling Method

Honeys from very different botanical origins were analyzed. The straightforward workflow developed is helpful for identifying differences in the bioactivity between monofloral honey types and can contribute to quality designation and branding. The effect-directed profiling provided new insights that are valuable for food science and nutrition. A wealth of 28 different bioactive compound zones were observed via effect-directed profiling. The effect-directed assays (Figure 1, Figure 2 and Figure 3) together with the HPTLC–HRMS analysis of selected zones (Figure 4, Figure 5 and Figure 6) thus revealed 69 different analytes of potential importance (Table 4). Since no data have been available in the literature on the identity of bioactive compounds in these nine different honey types studied, matching of HPTLC–HESI-HRMS signals and molecular formulas as well as tentative assignment to molecule structures was not possible. Nevertheless, a wealth of molecular formulas was successfully assigned due to the high-resolution feature of the MS system, and possible structural candidates were assigned (Table 4) according to the literature data from other honey types. This is how far one can go from effect-directed profiling to characterization of bioactive zones using the newly developed profiling method. In view of the number of samples and analytes, identification of all bioactive compound zones was not possible in the current study, which was mainly aimed at the development of bioactivity profiling.

For identification of the bioactive compounds, molecule fragmentation by HRMS/MS, co-chromatography with supposed standard substances, and nuclear magnetic resonance spectroscopy analyses would be further necessary options. For future structure elucidation, the separation system can be adjusted to the zone region of interest. For example, a very apolar mobile phase will better resolve the apolar compound zones (in comparison to our compromise-guided selection of the solvent system). After effect detection, the number of assignable mass signals in the recorded HRMS spectra from a better resolved zone should be reduced. As another option, a 2D separation using orthogonal mobile phases [37] or an orthogonal 8D hyphenation [22] can be studied for honey samples to differentiate the mass signals and to highlight the mass signals responsible for the individual effects.

## 3. Materials and Methods

### 3.1. Chemicals and Reagents

Methanol, ethanol, and 2-propanol (all HPLC grade), as well as formic acid and acetic acid (99%, LC–MS), were obtained from VWR, Darmstadt, Germany. Acetonitrile (HPLC grade) was received from Honeywell Specialty Chemicals, Seelze, Germany. Toluene (HPLC grade) was delivered by Promochem, LGC Standards, Wesel, Germany. Gram-negative, bioluminescent marine *Aliivibrio fischeri* bacteria (DSM–7151) were obtained from the German Collection of Microorganisms and Cell Cultures, Berlin, Germany. α-Glucosidase from *Saccharomyces cerevisiae* (1000 U/vial), α-amylase from hog pancreas (50 U/mg), 2-chloro-*p*-nitrophenyl-α-d-maltotrioside, acetylcholinesterase (AChE) from *Electrophorus electricus* (≥245 U/mg solid, 10 kU/vial), sodium acetate, di-sodium hydrogen phosphate, magnesium sulfate heptahydrate, sodium chloride, and ammonium hydroxide (>98) were delivered by Sigma–Aldrich, Steinheim, Germany. β-Glucosidase from almonds (3040 U/mg) and 2-naphthyl-β-d-glucopyranoside were provided by ABCR, Karlsruhe, Germany. Fast Blue B salt (95%) was from MP Biomedicals, Eschwege, Germany. 2,2-Diphenyl-1-picrylhydrazyl (DPPH•, 95%) was delivered by Alfa Aesar, Schwerte, Germany. 2-Naphtyl-α-d-glucopyranoside was from Fluorochem, Hadfield Derbyshire, UK. Tris(hydroxymethyl)aminomethane (Tris, ≥99.9%), dipotassium hydrogen phosphate (≥99%), sodium dihydrogen phosphate monohydrate (≥98%), sodium hydroxide (≥98%), hydrochloric acid (HCl, 37%), ethyl acetate (HPLC grade), and petroleum ether (40–60 °C) were purchased from Carl Roth, Karlsruhe, Germany. HPTLC silica gel 60 F_254_ plates (20 cm × 10 cm) were provided by Merck, Darmstadt, Germany. 1-Naphthyl acetate (≥98%) was obtained from AppliChem, Darmstadt, Germany. Bi-distilled water was produced with a Heraeus Destamat Bi–18E, Thermo Fisher Scientific, Dreieich, Germany. The polypropylene box (KIS, 27 cm × 16 cm × 10 cm, for plate incubation) was from ABM, Wolframs–Eschenbach, Germany. Honey samples were collected from the forest and honey-producing areas in the honey-harvesting period in Ethiopia, Africa.

### 3.2. Solid-Phase Extraction

Each honey sample (10 g) was dissolved in 100 mL of bi-distilled water, centrifuged (3000× *g*), and applied to spherical, hydrophobic polystyrene-divinylbenzene resin solid-phase extraction columns (CHROMABOND^®^ HR-X, 3 mL, 200 mg, 85 µm particle size, REF 730931) according to the product instruction Appl. No. 304310 (Macherey-Nagel, Düren, Germany). After elution with 10 mL methanol, the eluent solvent was concentrated to 1 mL, centrifuged (17,000× *g*, 5 min), and filled in a sampler vial for analysis.

### 3.3. HPTLC–UV/Vis/FLD Method

Each honey extract was applied (1–9 µL/band, as listed in the respective figure) in an 8 mm band (track distance 15 mm, distance from left edge 15 mm, distance from bottom edge 8 mm, and dosage speed 150 nL/s; Automatic TLC Sampler ATS 4, CAMAG, Muttenz, Switzerland), dried in a stream of cold air (2 min, hair dryer), and developed with ethyl acetate–methanol 3:2 (*V/V*) up to a migration distance of 60 mm (which took 14 min) in a twin trough chamber (20 × 10 cm, CAMAG), dried (3 min), and documented at white light illumination (Vis), UV 254 nm, and FLD 366 nm (TLC Visualizer, CAMAG). For derivatization, the plate was immersed in the respective reagent as described elsewhere [21,24,38,39]. Instruments were operated and data were processed with winCATS, version 1.4.7.2018, or visionCATS, version 3.1.21109.3 software (CAMAG).

### 3.4. Optional On-Surface Extraction and Separation on the Same Plate

This approach was only used for Section 2.5. Each honey sample (2 g) was diluted in 10 mL methanol, vortexed, ultrasonicated (15 min), and centrifuged (3000× *g*, 15 min). An aliquot of each supernatant was applied (25 µL) in an area of 8 mm *×* 10 mm (track distance 15 mm, distance from left edge 15 mm, distance from bottom edge 8 mm, and dosage speed 600 nL/s) and dried (2 min, stream of cold air). As an example of a combined on-surface extraction and separation on the same plate, the start areas were focused twice with acetonitrile–ethanol–ammonia 8:1:2, *V*/*V/V,* up to 30 mm, and after the plate was cut at 15 mm (TLC Plate Cutter, CAMAG) and developed with toluene–ethyl acetate–formic acid–water 1.6:7:0.8:0.6, *V*/*V/V*/*V*, up to a migration distance of 70 mm.

### 3.5. Effect-Directed Profiling

Six chromatograms were prepared in parallel. Depending on the assay, the sample volume was selected (listed in the figure caption) and respective positive controls were applied above the solvent front at the upper chromatogram edge [39]. Then, the plate was subjected to the respective assay solutions or suspensions. Each assay was repeated and the results were confirmed. For incubation in the incubator (at 37 °C, if not stated otherwise), each plate was placed horizontally in a humid polypropylene box (moistened a priori for 30 min with 35 mL water spread on filter papers aligned on walls and the bottom). Drying was performed in a stream of cold air (hair dryer) or on a TLC Plate Heater (CAMAG). If not stated otherwise, documentation was performed at white light illumination in the reflectance mode (TLC Visualizer, CAMAG).

#### 3.5.1. α- and β-Glucosidase Inhibition Assays

The chromatogram was piezoelectrically sprayed (green nozzle, level 6, Derivatizer, CAMAG) with 2 mL substrate solution (12 mg 2-naphthyl-α- (or β)-D-glucopyranoside in 9 mL ethanol and 1 mL 10 mmol sodium chloride solution) and dried (2 min). For prewetting, 1 mL sodium acetate buffer (10 g sodium acetate in 250 mL water, adjusted to pH 7.5 with 0.1 M acetic acid) was sprayed on the plate, followed by 2 mL enzyme solution (10 U/mL α-glucosidase, or 1000 U/mL β-glucosidase in buffer). The incubation took 15 min for α-glucosidase, or 30 min for β-glucosidase. Then, the plate was sprayed with 0.75 mL Fast Blue B salt solution (2 mg/mL in bi-distilled water) and dried [40,41]. Colorless (bright) spots on a purple background indicated α- or β-glucosidase inhibitors.

#### 3.5.2. Radical Scavenging Assay

The chromatogram was immersed (immersion speed 3.5 cm/s, immersion time 2 s) in a 0.05% methanolic DPPH• solution and dried (60 °C, 1 min) [42]. Image capture under white light illumination was carried out directly, but also repeated the next day, as the zone response increased over time. Radical scavenging compounds appeared as yellow zones against a purple background.

#### 3.5.3. *Aliivibrio fischeri* Bioassay

The chromatogram was immersed (immersion speed 3.5 cm/s and immersion time 2 s, TLC Immersion Device, CAMAG) in an *Aliivibrio fischeri* culture. The readiness of the bacterial growth prepared according to [43,44] was visually controlled for an emitted brilliant green-blue light by shaking the culture flask in a dark room. The bioluminescence was recorded with an exposure time of 50 s (time interval 3 min, BioLuminizer, CAMAG). Dark zones indicated antibacterial compounds.

#### 3.5.4. AChE Inhibition Assay

The AChE inhibition assay was performed as described [45,46]. The chromatogram was prewetted with 1 mL Tris-HCl buffer solution (pH 7.8, 0.05 M), then sprayed with 3 mL AChE solution (6.66 U/mL) and incubated at 37 °C for 25 min. The plate was sprayed with 0.75 mL of the substrate/chromogenic reagent mixture (ethanolic 1-naphthyl acetate solution 4.5 mg/1.5 mL and aqueous Fast Blue B salt solution, 9 mg/3 mL) and dried (3 min). Colorless (bright) spots on a purple background indicated AChE inhibitors.

#### 3.5.5. α-Amylase Inhibition Assay

The chromatogram was immersed in the substrate solution (1.4 mg/mL 2-chloro-*p*-nitrophenyl-α-D-maltotrioside in ethanol; immersion speed 3.5 cm/s and immersion time 2 s, TLC Immersion Device). The plate was dried (2 min), then immersed in the freshly prepared enzyme solution (1.2 mg/mL α-amylase in sodium acetate buffer) and incubated for 15 min at 37 °C [39]. Colorless (bright) spots on a yellow background indicated α-amylase inhibitors.

### 3.6. HPTLC–HESI–HRMS

The extracts (3 µL/band) or sample solutions (25 µL/area each) were applied in duplicate on the same HPTLC plate prewashed twice with methanol–water 3:1 (*V*/*V*) [47]. After development with toluene–ethyl acetate–formic acid–water 2:6:1:0.6, *V*/*V/V*/*V*, up to a migration distance of 70 mm, the plates were cut (smartCut Plate Cutter, CAMAG) into two identical halves; one was subjected to the DPPH• assay, and the other was kept clean for HRMS. The zones of interest were online eluted for 60 s with methanol at 0.2 mL/min into a Q Exactive Plus Hybrid Quadrupole Orbitrap system (Thermo Fisher Scientific, Dreieich, Germany) using an open-source modified fully automated elution head-based interface [48]. In between the interface and the mass spectrometer, a filter frit (Upchurch Scientific A-356 and PEEK frit Blue UPA-703, Techlab, Erkerode, Germany) was installed to prevent the HESI source from particles. The ionization settings were a spray voltage of 3.5 kV, capillary temperature of 270 °C, sheath gas of 20 (arbitrary units), aux gas of 10 (arbitrary units), probe heater temperature of 200 °C, and S-lens RF level of 50 (arbitrary units). Full scan spectra were recorded at a resolution of 280,000 (FWHM at *m/z* 200), AGC target 1e6, and maximum inject time 200 ms with lock masses 301.14103 (dibutyl phthalate, [M + H]^+^) and 413.26623 (diisoctyl phthalate, [M + H]^+^) in the positive and 112.98563 (formic acid, [2M+Na−2H]^−^) in the negative ionization mode. In between each analyte zone elution, the elution head and the tubing were cleaned via elution of a blank plate background (at a similar *hR*_F_ position as the target zone) to avoid cross-contamination. The blank spectrum was subtracted from the analyte mass spectrum. The instrument was controlled and the data were processed with Xcalibur 4.2.47 SP1 with Foundation 3.1.261.0 SP6 and SII for Xcalibur 1.5.0.10747 (Thermo Fisher Scientific).

## 4. Conclusions

The developed non-target effect-directed profiling of nine different types of monofloral Ethiopian honey using HPTLC−UV/Vis/FLD−EDA provided new insights that are helpful for the valorization of Ethiopian honeys. Highlighting the physiological activity of Ethiopian honeys based on bioactivity profiles is valuable for food science and nutrition as well as for the market. It is important to understand the differences in the bioactivity profiles between monofloral honeys since it can affect quality designation and branding. The effect-directed profiles of radical scavenging compounds, antibacterial compounds against Gram-negative bacteria, and compounds inhibiting AChE, α-glucosidase, β-glucosidase, and α-amylase, contributed to the differentiation, categorization, and authentication of the honey types. Given the global trade in honey and the associated vulnerability to fraud, contamination, and pesticide or antibiotic residues, non-target profiling is also able to detect other bioactive ingredients that have not previously been in focus. The non-target effect-directed profiling developed could provide more comprehensive information on honey quality and safety compared to conventional target analysis or microtiter plate assays, which only provide a sum parameter. It was also shown that the further characterization of bioactive zones of interest and the assignment of molecular structures was possible using HPTLC–HESI-HRMS, although structural assignment was limited.

## Figures and Tables

**Table 1:** Harvest period and Ethiopian sampling site of the collected floral honey samples with respective color depicted (ID numbers indicate individual samples taken).

Honey Color	ID	Floral Honey Source	Pollen Count (Out of 500)	Pollen Dominancy (%)	Harvested in 2018	Sampling Site
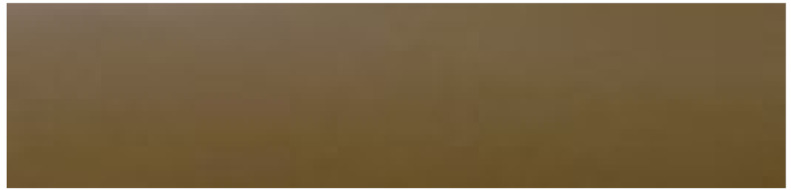	A	*Acacia* spp.	310	62	September, October	Zikwala
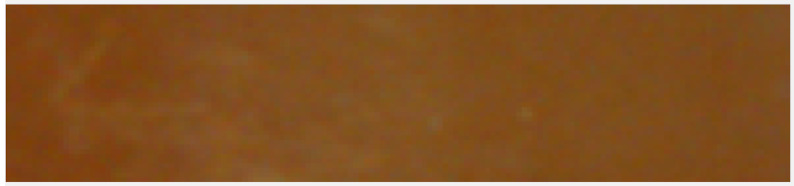	B1	*Becium grandi* *fl* *orum*	370	74	August, September	Maychew
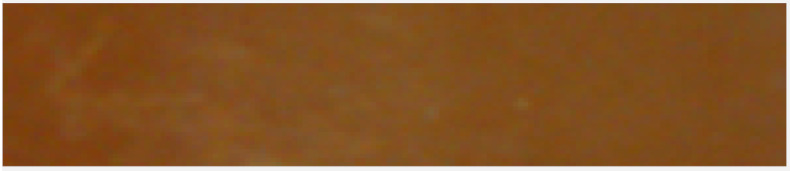	B2					Wukro
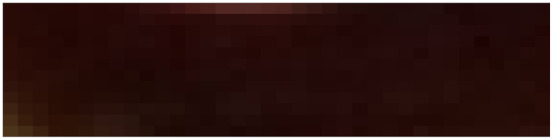	C1/2	*Croton macrostachyus*	300	60	June, July	Dello Mena
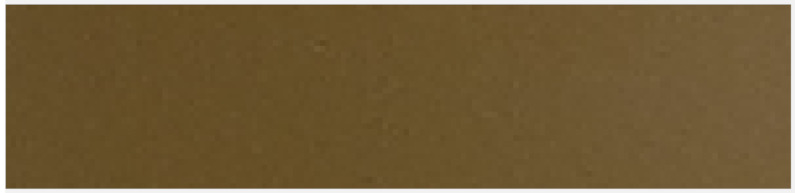	E	*Eucalyptus globulus*	430	86	April, May, June	Addis Ababa, Yeka
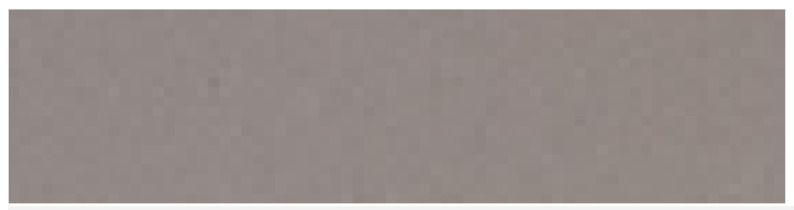	H	*Hypoestes* spp.	315	63	September, October	Wukro
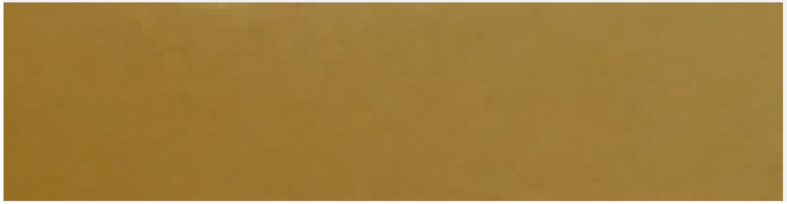	L	*Leucas abyssinica*	430	86	September, October	Maychew
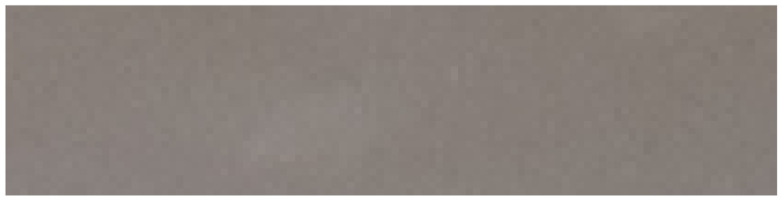	S	*Schef* *fl* *era abyssinica*	450	90	April, May	Sheka, Bonga
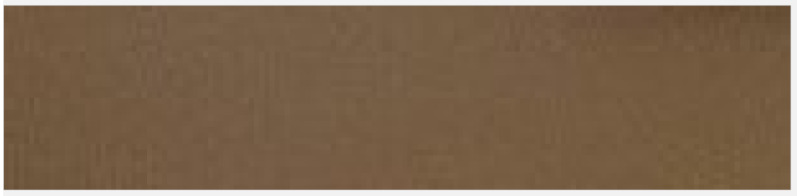	V	*V* *ernonia amygdalina*	390	78	January, February	Becho, Anfillo, Gida-Ayana
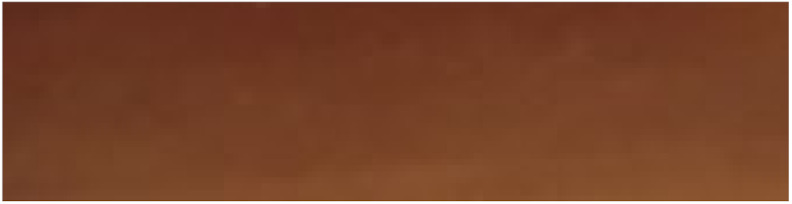	Y1–4	*Coffea* *arabica*		75	February, March	Yayu
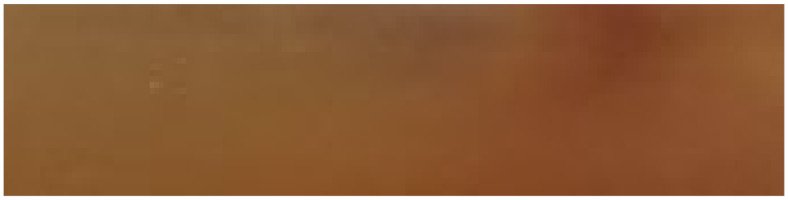	G1–4		375			Goma
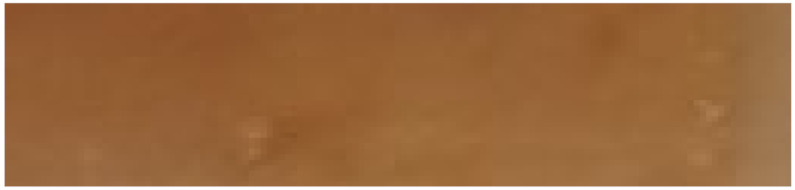	M1–4					Mana

**Table 2:** Main bioactive zones **1**–**28** (Figure 1, Figure 2 and Figure 3) observed in the monofloral honeys (as assigned in Table 1; honey with pronounced effect in bold).

Zone	Radical Scavenger	Glucosidase Inhibitor	AChE Inhibitor	Amylase Inhibitor *	Antibacterial
1		A **C V YGM**			
2		C **YGM**			
3		C			
4		C			
5		C			
6		A B **C S V**			
7					C
8					C YGM
9					C
10					**C**
11					B L YGM
12					C V YGM
13	A B **C E V YGM**				**C E V YGM**
14	S				S
15	**A B C E S V YGM**		C YGM		
16	C		C		
17	C		C		
18	B C E S V				
19	S V **YGM**				
20			C **YGM**		
21			**C YGM**		
22			E		
23			E		
24			V		
25				A B **C E** S V	
26			**YGM**		
27			**YGM**		
28	**YGM**				

* Assay not performed for *Coffea arabica* (Y, G, M).

**Table 3:** On-surface extraction and separation on the same plate: Investigated solvent systems on HPTLC silica gel 60 F_254_ plate for focusing of the *Coffea arabica* honey applied as an area to a sharp start band and for subsequent separation of the compounds, as well as an example of a combination.

Focusing of the Start Area	Ratio (*V*/*V*)	Chromatogram at UV/Vis/FLD
Methanol	1	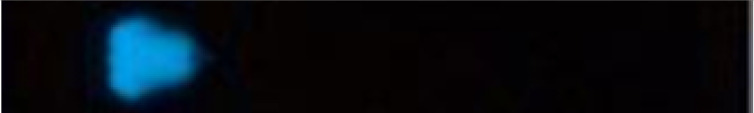
Acetonitrile	1	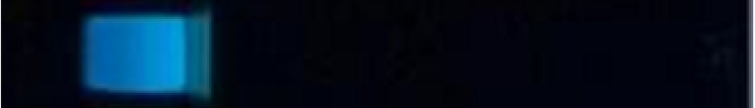
2-Propanol	1	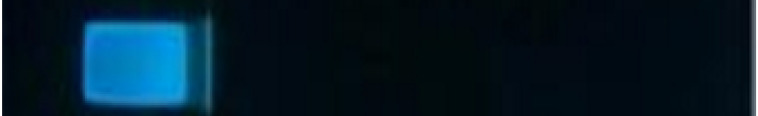
Acetonitrile–methanol–ammonium hydroxide	5:3:2	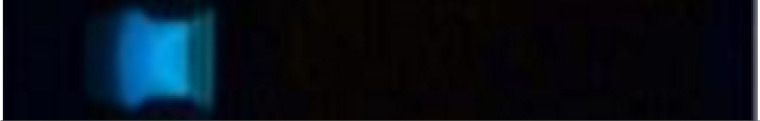
Acetonitrile–ethanol–ammonium hydroxide	8:1:1	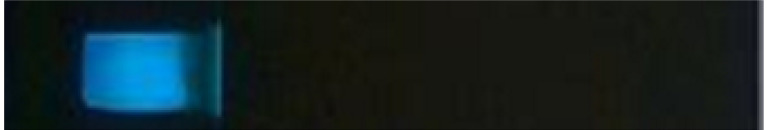
Acetonitrile–ethanol–ammonium hydroxide	1:3:1	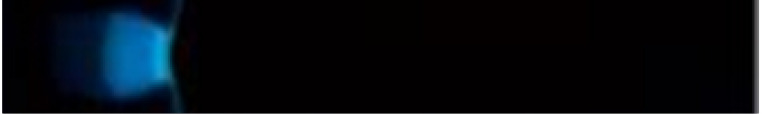
Acetonitrile–water	9:1	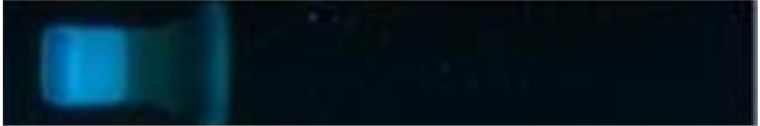
Ethanol–ammonium hydroxide	9:1	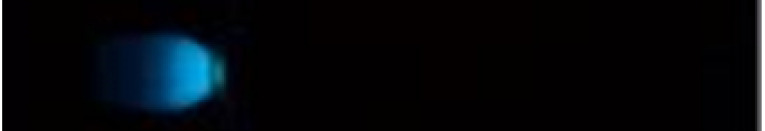
Methanol–ammonium hydroxide	10:1	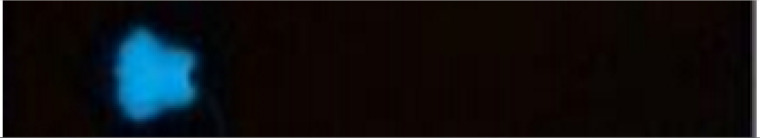
Ethanol–formic acid–water	7:1:2	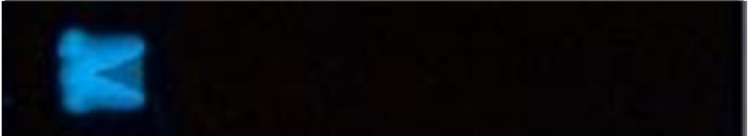
**Mobile Phase System for Separation**		
Ethyl acetate–ethanol–water	3.8:1:0.2	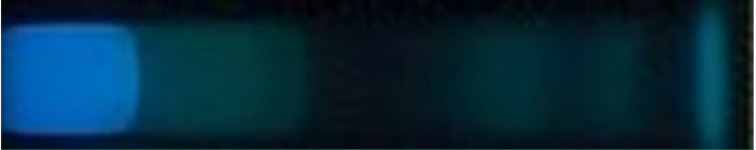
Toluene–ethyl acetate–ethanol	2.4:1.8:0.8	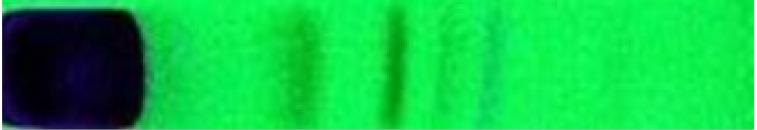
Petroleum ether–ethyl acetate–acetone	3.8:0.6:0.7	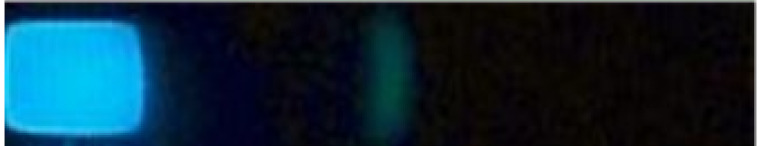
Ethyl acetate–ethanol–ammonium hydroxide	4.2:0.6:0.2	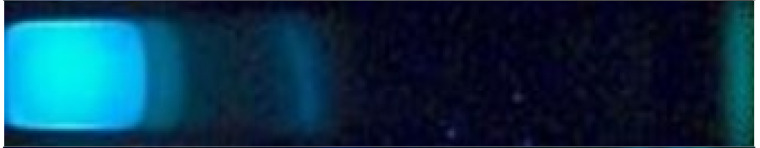
Toluene–ethyl acetate–formic acid–water	2:6:1:0.6	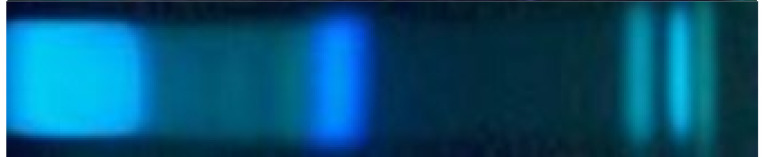
Toluene–ethyl acetate–formic acid–water	1.6:7:0.8:0.6	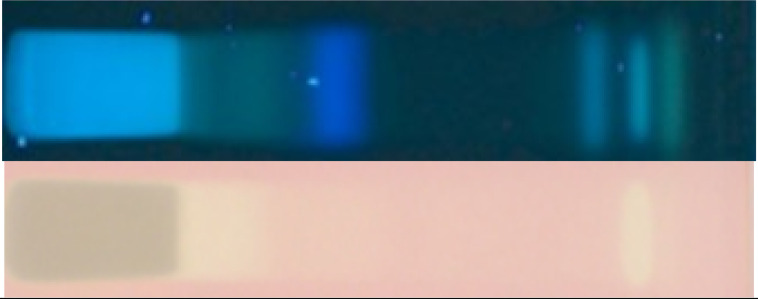
**On-Surface Extraction and Separation**		
1. Focusing twice with acetonitrile–ethanol–ammonia to 3 cm, then plate cut at 1.5 cm	8:1:2	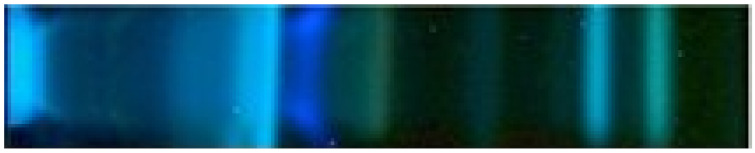
2. Separation with toluene–ethyl acetate–formic acid–water	1.6:7:0.8:0.6	

**Table 4:** Complete list of analytes found in the various honey samples and their tentative assignment to possible structures according to the literature.

Zone	Mass Signal *m*/*z*	Adduct	Molecular Formula (Neutral Molecule)	Δ ppm	Tentative Assignment According to the Literature	Lit.
**18 (B1),** **Figure 4**	389.1780	[M1 + Na]^+^	C_16_H_30_O_9_	0.50		
	547.1999	[M2 + Na]^+^	C_22_H_36_O_14_	−0.29		
	523.2045	[M2 − H]^−^		−3.26		
	209.0823	[M3 − H]^−^	C_11_H_14_O_4_	−2.61		
	342.1201	[M4 − H]^−^	Unknown	-		
	621.1722	[M5 − H]^−^	C_23_H_42_O_13_S_3_	−0.76		
**13 (C1)**, **Figure 4**	146.0601	[M6 + H]^+^	C_9_H_7_NO	−0.62	2-Hydroxyquinoline in various Iranian honeys	[31]
	144.0456			−0.71		
	199.0578	[M7 + Na]^+^	C_7_H_12_O_5_	−0.47		
	213.0734	[M8 + Na]^+^	C_8_H_14_O_5_	−0.16		
	263.1254	[M9 + Na]^+^	C_13_H_20_O_4_	−0.07		
	289.1538	[M10 + Na]^+^	Unknown	-		
	171.0566	[M11 − H] ^−^	C_10_H_8_N_2_O	−1.01		
	179.0351	[M12 − H] ^−^	C_9_H_7_O_4_	−0.87	Caffeic acid in various Iranian honeys	[31]
	190.0511	[M13 − H] ^−^	C_10_H_9_NO_3_	−0.85		
	199.0515	[M14 − H] ^−^	C_11_H_8_N_2_O_2_	−0.89		
**13 (E)**, **Figure 4**	209.0821	[M3 − H] ^−^	C_11_H_14_O_4_	−1.27		
	289.1534	[M10 + Na] ^+^	Unknown	-		
	187.0728	[M15 + H] ^+^	C_10_H_12_O_2_	0.91		
	207.1376	[M16 + Na] ^+^	C_13_H_19_O_2_	1.48		
	237.1095	[M17 + Na] ^+^	C_11_H_18_O_4_	1.19		
	223.0938	[M18 + Na] ^+^	C_10_H_16_O_4_	1.13	Succinic acid monocyclohexyl ester in various Iranian honeys	[31]
					(*E*)-2-Decenedioic acid in various Italian honeys	[32]
	247.1301	[M19 + Na] ^+^	C_13_H_20_O_3_	1.48		
	497.2140	[M20 + Na] ^+^	C_26_H_34_O_8_	1.15		
**14 (S)**, **Figure 4**	211.1303	[M21 + Na] ^+^	C_10_H_20_O_3_	−1.05	Hydroxy decenoic acid in various Iranian honeys	[31]
	399.2714	[2M21 + Na] ^+^		0.88		
	187.1341	[M21 − H] ^−^		0.75		
	197.0821	[M22 − H] ^−^	C_10_H_14_O_4_	−0.74		
	211.0614	[M23 − H] ^−^	C_10_H_12_O_5_	−1.05	Methyl syringate in various Iranian hones	[31]
	242.0674	[M24 − H] ^−^	C_10_H_13_NO_6_	−1.39		
	361.1511	[M25 − H] ^−^	C_17_H_29_O_4_S_2_	0.60		
	459.1187	[M26 − H] ^−^	C_16_H_28_O_13_S	−2.00		
**15 (V)**, **Figure 4**	190.1229	[M27 + H] ^+^	C_12_H_15_NO	−1.16		
	236.1284	[M28 + H] ^+^	C_13_H_17_NO_3_	−1.31		
	234.1145	[M28 − H] ^−^		−3.94		
	266.1389	[M29 + H] ^+^	C_14_H_19_NO_4_	−0.51		
	264.1251	[M29 − H] ^−^		−3.74		
	289.1184	[M30 + H] ^+^	C_15_H_16_N_2_O_4_	−0.64		
	342.1549	[M31 + H] ^+^	C_16_H_23_NO_7_	−0.53		
**11 (G)**, **Figure 5**	183.0993	[M32 + Na] ^+^	C_8_H_16_O_3_	−0.84		
	159.1028	[M32 − H] ^−^		−0.51		
	209.1149	[M33 + Na] ^+^	C_10_H_18_O_3_	−0.59	Royal jelly acid in various Iranian honeys	[31]
	185.1184	[M33 − H] ^−^		−0.49		
	227.1254	[M34 + Na] ^+^	C_10_H_20_O_4_	−0.22		
	243.0741	[M35 + H] ^+^	Unknown	-		
	267.1104	[M36 + H] ^+^	Unknown	-		
	293.0995	[M37 + Na] ^+^	C_13_H_18_O_6_	0.14		
	321.0945	[M38 + Na] ^+^	C_14_H_18_O_7_	−0.11		
	355.1727	[M39 + Na] ^+^	C_16_H_28_O_7_	0.07		
	371.1677	[M40 + Na] ^+^	C_16_H_28_O_8_	−0.13		
	376.1003	[M41 + H] ^+^	C_15_H_19_O_11_	−0.70		
	121.0296	[M42 − H] ^−^	C_7_H_6_O_2_	−0.55	Benzoic acid in honeydew honey from Brazil	[33]
	151.0402	[M43 − H] ^−^	C_8_H_8_O_3_	−0.60	Mandelic acid in various honeys	[34]
					Vanillin in various Czech honeys	[35]
	240.0516	[M44 − H] ^−^	C_10_H_11_NO_6_	−1.03		
	279.1241	[M45 − H] ^−^	C_15_H_20_O_5_	−0.90		
**12 (G)**, **Figure 5**	211.1305	[M21 + Na] ^+^	C_10_H_20_O_3_	−0.26	Hydroxy decanoic acid in various Iranian honeys	[31]
	209.1150	[M33 + Na] ^+^	C_10_H_18_O_3_	−0.93	Royal jelly acid in various Iranian honeys	[31]
	151.0401	[M43 − H] ^−^	C_8_H_8_O_3_	−0.21	Mandelic acid in various honeys	[34] [35]
					Vanillin in various Czech honeys	
	171.0992	[M46 + H] ^+^	C_7_H_16_O_3_	−0.43		
	188.0683	[M47 + H] ^+^	C_8_H_11_O_5_	−2.04		
	287.1254	[M48 + Na] ^+^	C_15_H_20_O_4_	0.04		
	263.1290	[M48 − H] ^−^				
	137.0244	[M49 − H] ^−^	C_7_H_6_O_3_	−0.15	Salicylic acid in honeydew honey from Brazil	[33]
					4-Hydroxybenzoic acid in *Agastache* honey	[36]
**13 (G)**, **Figure 5**	146.0600	[M6 + Na] ^+^	C_9_H_7_NO	0.07	2-Hydroxyquinoline in various Iranian honeys	[31]
	263.1252	[M9 + Na] +	C_13_H_20_O_4_	0.76		
	247.1303	[M19 + Na] ^+^	C_13_H_20_O_3_	0.71		
	170.0576	[M50 + Na] ^+^	C_9_H_9_NO	0.26		
	146.0612	[M50 − H] ^−^		0.07		
	193.1199	[M51 + Na] ^+^	C_10_H_28_O_2_	0.21		
**15 (G)**, **Figure 5**	105.0702	[M52 + H] ^+^	C_8_H_8_	−3.37		
	122.0966	[M53 + H] ^+^	Unknown	-		
	203.0525	[M54 + Na] ^+^	C_6_H_12_O_6_	0.05	Hexose	
	258.1099	[M55 + H] ^+^	C_12_H_17_O_6_	−0.89		
	284.1490	[M56 + H] ^+^	Unknown	-		
	211.0613	[M57–H] ^−^	C_10_H_12_O_5_	−0.24	Methyl syringate in various Iranian honeys	[31]
**19 (G)**, **Figure 5**	146.0600	[M6 + H] ^+^	C_9_H_7_NO	0.07	2-Hydroxyquinoline in various Iranian honeys	[31]
	168.0419	[M6 + Na] ^+^		0.15		
	144.0455	[M6 − H] ^−^		−0.01		
	195.0876	[M58 + H] ^+^	C_18_H_10_N_4_O_2_	0.27		
	217.0695	[M58 + Na] ^+^		0.22		
	291.1125	[M59 + H] ^+^	C_18_H_14_N_2_O_2_	0.67		
	313.0945	[M59 + Na] ^+^		0.67		
	201.1133	[M60 − H] ^−^	C_10_H_18_O_4_	−0.23	Decanedioic acid in various Italian honeys	[32]
**28 (G)**, **Figure 5**	146.0601	[M6 + H] ^+^	C_9_H_7_NO	−1.57	2-Hydroxyquinoline in various Iranian honeys	[31]
	168.0421	[M6 + Na] ^+^		−1.52		
	144.0455	[M6 − H] ^−^		−0.22		
	214.0475	[M13 + Na] ^+^	C_10_H_9_NO_3_	−1.14		
	190.0510	[M13 − H] ^−^		−0.22		
	146.0612	[M50 − H] ^−^	C_9_H_9_NO	−0.22		
	236.0294	[M61 + Na] ^+^	C_9_H_9_O_6_	−2.05		
	359.1311	[M62 + Na] ^+^	C_14_H_24_O_9_	−0.68		
	385.1468	[M63 + Na] ^+^	C_16_H_26_O_9_	−0.82		
	188.0355	[M64 − H] ^−^	C_10_H_7_NO_3_	−1.02		
**I (V)**, **Figure 6**	146.0601	[M6 + H] ^+^	C_9_H_7_NO	−1.57	2-Hydroxyquinoline in various Iranian honeys	[31]
	168.0421	[M6 + Na] ^+^		−1.52		
	144.0455	[M6 − H] ^−^		−0.22		
	261.1098	[M65 + Na] ^+^	C_13_H_18_O_4_	0.00		
	237.1133	[M65 − H] ^−^		−0.40		
	304.2610	[M66 + Na] ^+^	C_18_H_35_NO	0.31		
	253.1083	[M67 − H] ^−^	C_13_H_18_O_5_	−0.40		
**II (Y2)**, **Figure 6**	146.0601	[M6 + H] ^+^	C_9_H_7_NO	−0.48	2-Hydroxyquinoline in various Iranian honeys	[31]
	168.0421	[M6 + Na] ^+^		−0.33		
	313.0946	[2M6 + Na] ^+^		0.35		
	144.0455	[M6 − H] ^−^		0.06		
**III (V)**, **Figure 6**	146.0601	[M6 + H] ^+^	C_9_H_7_NO	−0.48	2-Hydroxyquinoline in various Iranian honeys	[31]
	144.0455	[M6 − H] ^−^		0.06		
	263.1253	[M9 + Na] ^+^	C_13_H_20_O_4_	0.12		
	247.1304	[M19 + Na] ^+^	C_13_H_20_O_3_	0.27		
	209.1148	[M33 + Na] ^+^	C_10_H_18_O_3_	−0.02	Royal jelly acid in various Iranian honeys	[31]
	281.1358	[M68 + Na] ^+^	C_13_H_22_O_5_	0.41		
**IV (V)**, **Figure 6**	144.0455	[M6 − H] ^−^	C_9_H_7_NO	0.12	2-Hydroxyquinoline in various Iranian honeys	[31]
	263.1255	[M9 + Na] ^+^	C_13_H_20_O_4_	0.12		
	211.1306	[M21 + Na] ^+^	C_10_H_20_O_3_	−0.21	Hydroxy decenoic acid in various Iranian honeys	[31]
	399.2719	[2M21 + Na] ^+^		−0.05		
**V (Y2)**, **Figure 6**	146.0601	[M6 + H] ^+^	C_9_H_7_NO	−0.48	2-Hydroxyquinoline in various Iranian honeys	[31]
	144.0455	[M6 − H] ^−^		0.12		
	203.0527	[M69 + Na] ^+^	C_6_H_12_O_6_	−0.39	Hexose	

## Data Availability

The data presented in this study are available on request from the corresponding author.

## Sample Availability

Samples are available on request.
